# Differential roles of STAT3 depending on the mechanism of STAT3 activation in gastric cancer cells

**DOI:** 10.1038/bjc.2011.246

**Published:** 2011-07-05

**Authors:** W Okamoto, I Okamoto, T Arao, K Yanagihara, K Nishio, K Nakagawa

**Affiliations:** 1 Department of Medical Oncology, Kinki University Faculty of Medicine, 377-2 Ohno-higashi, Osaka-Sayama, Osaka 589-8511, Japan; 2 Department of Genome Biology, Kinki University Faculty of Medicine, 377-2 Ohno-higashi, Osaka-Sayama, Osaka 589-8511, Japan; 3 Laboratory of Health Sciences, Department of Life Sciences, Faculty of Pharmacy, Yasuda Women's University, 6-13-1 Yasuhigashi, Asaminami, Hiroshima 731-0153, Japan

**Keywords:** STAT3, MET, IL-6, JAK, apoptosis, migration and invasion

## Abstract

**Background:**

Signal transducer and activator of transcription 3 (STAT3) is a transcription factor that is activated in response to growth factors and cytokines, and which contributes to the regulation of cell proliferation, apoptosis, and motility in many human tumour types.

**Methods:**

We investigated the mechanisms of STAT3 activation and the function of STAT3 depending on its mechanism of activation in gastric cancer cells.

**Results:**

The MET-tyrosine kinase inhibitor (TKI) and cell transfection with a small interfering RNA (siRNA) specific for MET mRNA inhibited STAT3 phosphorylation in MET-activated cells, indicating that STAT3 activation is linked to MET signalling. Forced expression of a constitutively active form of STAT3 also attenuated MET-TKI-induced apoptosis, suggesting that inhibition of STAT3 activity contributes to MET-TKI-induced apoptosis. MKN1 and MKN7 cells, both of which are negative for MET activation, produced interleukin-6 (IL-6) that activated STAT3 through the Janus kinase pathway. Depletion of STAT3 by siRNA inhibited migration and invasion of these cells, suggesting that STAT3 activated by IL-6 contributes to regulation of cell motility.

**Conclusion:**

Our data thus show that activated STAT3 contributes to either cell survival or motility in gastric cancer cells, and that these actions are related to different mechanisms of STAT3 activation.

Gastric cancer is the second most frequent cause of cancer deaths worldwide (Hartgrink *et al*, 2009). Chemotherapy has a beneficial effect on survival in individuals with advanced-stage gastric cancer, but even so overall survival is usually still only ∼1 year (Hartgrink *et al*, 2009; Wesolowski *et al*, 2009). Substantial advances in the development of molecularly targeted therapies for gastric cancer have been achieved in recent years. Amplification of the proto-oncogene *MET* is a frequent (10–20%) molecular abnormality in gastric cancer (Kuniyasu *et al*, 1992; Nessling *et al*, 1998; Sakakura *et al*, 1999), and a MET-tyrosine kinase inhibitor (TKI) has been shown to induce apoptosis in gastric cancer cells with *MET* amplification (Smolen *et al*, 2006; Okamoto *et al*, 2010). However, the downstream mediators of MET-TKI-induced apoptosis in *MET* amplification-positive cells have remained unknown.

Signal transducer and activator of transcription (STAT) proteins were identified as transcription factors that are activated in response to exposure of cells to growth factors or cytokines (Lim and Cao, 2006). Activation of STAT3 mediates regulation of cell proliferation, apoptosis, migration, and differentiation in various cell types, and it is induced by the cytokine receptor-associated Janus kinase (JAK) family of nonreceptor tyrosine kinases or by receptor tyrosine kinases. Although STAT3 is often expressed at a high level and is constitutively activated in many human tumours (Garcia *et al*, 2001; Dhir *et al*, 2002; Song *et al*, 2003; Kanda *et al*, 2004; Silver *et al*, 2004), the mechanisms of STAT3 activation have not been completely characterised in gastric cancer. We have therefore now investigated whether activation of MET contributes to STAT3 activation and further examined the roles of STAT3 in human gastric cancer cell lines.

## Materials and methods

### Cell culture and reagents

The human gastric cancer cell lines SNU5 and N87 were obtained from American Type Culture Collection (Manassas, VA, USA); MKN1, MKN7, MKN45, and AZ521 were from the Health Science Research Resources Bank (Osaka, Japan); OKAJIMA and MKN28 were from Immuno-Biological Laboratories (Gunma, Japan); and SNU216 was from the Korean Cell Line Bank (Seoul, Korea). HSC58, 58As1, and 58As9 are established cell lines derived from human scirrhous gastric carcinoma, as previously described (Yanagihara *et al*, 2005). All cells were cultured under a humidified atmosphere of 5% CO_2_ at 37°C in RPMI 1640 medium (Sigma, St Louis, MO, USA) supplemented with 10% fetal bovine serum and were passaged for ⩽3 months before renewal from frozen, early-passage stocks obtained from the indicated sources. Cells were regularly screened for mycoplasma with the use of a MycoAlert Mycoplasma Detection Kit (Lonza, Rockland, ME, USA). PHA-665752 was obtained from Tocris Bioscience (Bristol, UK), pyridone 6 was from Calbiochem (La Jolla, CA, USA), and B-E8 was from Diaclone (Besancon, France).

### Immunoblot analysis

Cells were washed twice with ice-cold phosphate-buffered saline and then lysed with 1 × Cell Lysis Buffer (Cell Signaling Technology, Danvers, MA, USA), which contains 20 mM Tris-HCl (pH 7.5), 150 mM NaCl, 1 mM EDTA (disodium salt), 1 mM EGTA, 1% Triton X-100, 2.5 mM sodium pyrophosphate, 1 mM *β*-glycerophosphate, 1 mM Na_3_VO_4_, leupeptin (1 *μ*g ml^−1^), and 1 mM phenylmethylsulphonyl fluoride. The protein concentration of cell lysates was determined with a BCA protein assay kit (Thermo Fisher Scientific, Rockford, IL, USA), and equal amounts of protein were subjected to SDS-polyacrylamide gel electrophoresis on 7.5 or 12% gels (Bio-Rad, Hercules, CA, USA). The separated proteins were transferred to a nitrocellulose membrane, which was then incubated with Blocking One solution (Nacalai Tesque, Kyoto, Japan) for 20 min at room temperature before incubation overnight at 4°C with primary antibodies. Antibodies to phosphorylated STAT3 (phospho-Tyr^705^), total STAT3, phosphorylated MET (phospho-Tyr^1234/1235^), caspase-3, poly(ADP-ribose) polymerease (PARP), E-cadherin, or FLAG were obtained from Cell Signaling Technology; those to total MET were from Zymed/Invitrogen (Carlsbad, CA, USA); and those to *β*-actin were from Sigma. The membrane was then washed with phosphate-buffered saline containing 0.05% Tween-20 before incubation for 1 h at room temperature with horseradish peroxidase-conjugated antibodies to rabbit or mouse immunoglobulin G (GE Healthcare, Little Chalfont, UK). Immune complexes were finally detected with ECL Western Blotting Detection Reagents (GE Healthcare).

### Cell transfection

A pBabe-puro vector encoding STAT3-CA with a COOH-terminal FLAG tag was kindly provided by J Bromberg (Bromberg *et al*, 1999). Retroviruses encoding STAT3-CA were produced and used to infect OKAJIMA and 58As9 cells as described (Tanaka *et al*, 2009). Cells stably expressing STAT3-CA were isolated by selection with puromycin (InvivoGen, San Diego, CA, USA) at 1 *μ*g ml^−1^.

### DNA fragmentation assay

DNA fragmentation was quantified with the use of a Cell Death Detection ELISA Plus kit (Roche Applied Science, Indianapolis, IN, USA). In brief, cells (1 × 10^4^) isolated by centrifugation were lysed by incubation for 30 min at room temperature in 200 *μ*l of lysis buffer. The lysates were centrifuged at 200 **g** for 10 min, and portions (20 *μ*l) of the resulting supernatants were transferred to wells of the microplate and mixed with 80 *μ*l of immunoreagent. The plate was incubated at room temperature for 2 h with gentle shaking, after which the wells were washed three times, ABTS solution was added, and the colour reaction was developed. Stop solution was added to the wells, and the absorbance at 405 nm was measured (reference wavelength of 490 nm) with the use of a Multiskan Spectrum instrument (Thermo Labsystems, Boston, MA, USA). Samples were analysed in triplicate.

### Interleukin-6 ELISA

Cells (3 × 10^5^ per well) were seeded in six-well plates, cultured overnight in complete medium, and then incubated in serum-free medium for 24 h, after which the latter medium was collected and assayed for interleukin-6 (IL-6) with the use of a Human IL-6 Quantikine ELISA Kit (R&D Systems, Minneapolis, MN, USA). A standard curve was generated with the supplied reagents, and IL-6 concentration was determined as the average from triplicate samples.

### Gene silencing

Cells were plated at 50–60% confluence in six-well plates and incubated for 24 h before transient transfection with small interfering RNAs (siRNAs) for 24 or 48 h with the use of the Lipofectamine RNAiMAX reagent (Invitrogen). The siRNAs specific for human STAT3 mRNA (5′-UCAUUGACCUUGUGAAAAA-3′) or human MET mRNA (5′-ACAAGAUCGUCAACAAAAATT-3′), as well as a nonspecific siRNA (control), were obtained from Nippon EGT (Toyama, Japan). The cells were then subjected to immunoblot analysis or assays of cell migration and invasion.

### Cell migration and invasion assays

Cell migration and invasion were measured with BD BioCoat migration or Matrigel invasion assays (Becton Dickinson Labware, Bedford, MA, USA), respectively. Cells were isolated by exposure to trypsin, resuspended in serum-free RPMI 1640 medium, and transferred (1 × 10^5^ cells in 500 *μ*l) to the upper chamber, and 750 *μ*l of medium containing 10% fetal bovine serum were added to the lower chamber. After overnight incubation, cells remaining on the upper surface of the filter were removed with a cotton swab, whereas cells that had migrated or invaded to reach the lower surface were fixed, stained with a Diff Quick Staining Set (Sysmex, Kobe, Japan), and counted with the use of a light microscope ( × 40 magnification).

### Statistical analysis

Quantitative data are presented as means±s.d. and were analysed with Student's two-tailed *t*-test. A *P*-value of <0.05 was considered statistically significant.

## Results

### Activation of STAT3 is associated with MET activation in gastric cancer cell lines

**Figure 1:**
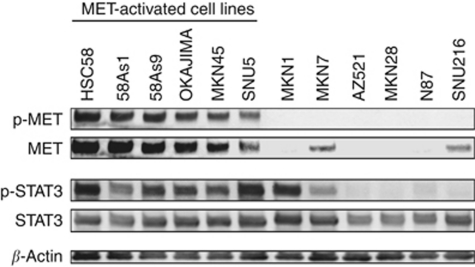
Phosphorylation of MET and STAT3 in gastric cancer cell lines. The indicated gastric cancer cell lines maintained in medium containing 10% serum were lysed and subjected to immunoblot analysis with antibodies to phosphorylated (p-) or total forms of MET or STAT3 or to *β*-actin (loading control).

We first examined the baseline levels of total STAT3 and activated (Tyr^705^-phosphorylated) STAT3 in 12 human gastric cancer cell lines by immunoblot analysis. All the cell lines expressed STAT3, whereas eight of the lines manifested detectable levels of STAT3 phosphorylation (Figure 1). We also determined the activation status of MET, as reflected by phosphorylation of tyrosine residues 1234 and 1235. Immunoblot analysis revealed that all six cell lines manifesting MET activation also showed high levels of STAT3 activation, whereas the remaining cell lines negative for MET activation, with the exception of MKN1 and MKN7, had undetectable levels of activated STAT3.

**Figure 2:**
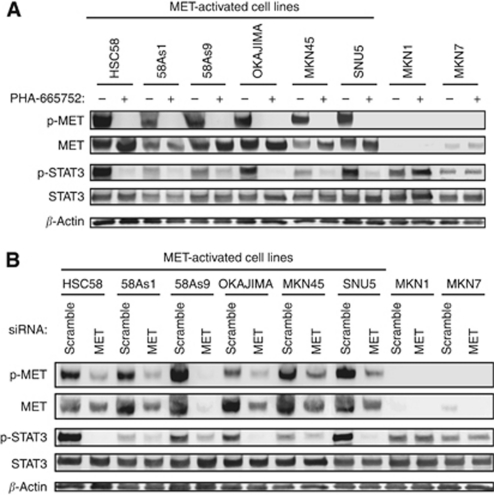
Effects of inhibition or depletion of MET on intracellular signalling in gastric cancer cell lines. (**A**) The indicated cell lines were incubated in medium containing 10% serum for 24 h in the absence or presence of 500 nM PHA-665752. Cell lysates were then subjected to immunoblot analysis with antibodies to phosphorylated (p-) or total forms of MET or STAT3 or to *β*-actin. (**B**) Cells were transfected with nonspecific (scramble) or MET siRNAs for 48 h, after which cell lysates were subjected to immunoblot analysis as in A.

To examine whether the activation of MET contributes to STAT3 activation, we determined the effects of the MET-TKI PHA-665752 on the phosphorylation of STAT3 in gastric cancer cells with STAT3 activation. PHA-665752 inhibited activation of STAT3 together with that of MET in MET-activated cell lines (Figure 2A). However, PHA-665752 had no effect on STAT3 phosphorylation in MKN1 and MKN7 cells, which are negative for MET activation. We also examined the effects of RNA interference (RNAi)-mediated depletion of MET. Similar to the effects of PHA-665752, transfection with a siRNA specific for MET mRNA resulted in inhibition of STAT3 phosphorylation in gastric cancer cells with MET activation but not in MKN1 or MKN7 cells (Figure 2B). These data thus suggested that activated MET induces STAT3 activation in gastric cancer cells with MET activation, whereas STAT3 is activated independently of MET in cells without MET activation.

### Role of STAT3 in PHA-665752-induced apoptosis in MET-activated gastric cancer cells

**Figure 3:**
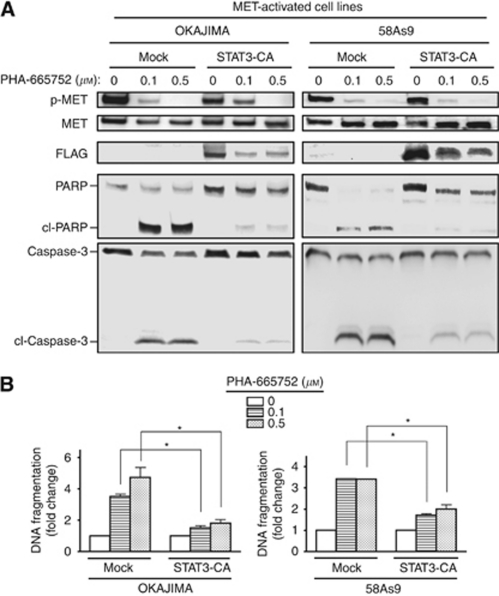
Role of STAT3 in MET-TKI-induced apoptosis in gastric cancer cells. (**A**) OKAJIMA or 58As9 cells infected with a retrovirus encoding FLAG-tagged STAT3-CA or with the empty virus (mock) were incubated in medium containing 10% serum for 48 h in the absence or presence of PHA-665752 at 0.1 or 0.5 *μ*M. Cell lysates were then subjected to immunoblot analysis with antibodies to total or phosphorylated forms of MET, FLAG, PARP, or caspase-3. The positions of full-length and cleaved (cl-) forms of PARP and caspase-3 are indicated. (**B**) Cells treated as in A were assayed for DNA fragmentation as described in Materials and Methods. Data are expressed as fold change relative to the corresponding value for mock infected cells not exposed to PHA-665752 and are means±s.d. from three independent experiments. ^*^P<0.05 for the indicated comparisons.

We and others previously showed that the MET-TKI induced a substantial increase in the frequency of apoptosis in MET-activated gastric cancer cells (Smolen *et al*, 2006; Okamoto *et al*, 2010). Consistent with these results, PHA-665752 induced cleavage of both caspase-3 and PARP as well as DNA fragmentation, indicative of the induction of apoptosis, in OKAJIMA and 58As9 cell lines, both of which are positive for MET activation (Figure 3). Given that PHA-665752 inhibited STAT3 phosphorylation and induced apoptosis, we investigated the possible role of STAT3 in PHA-665752-induced apoptosis by transfecting MET-activated cells with an expression vector encoding a FLAG epitope-tagged constitutively active (CA) form of human STAT3. We found that expression of STAT3-CA inhibited the cleavage of both caspase-3 and PARP, as well as DNA fragmentation triggered by PHA-665752 (Figure 3). These data thus suggested that inhibition of STAT3 activation contributes to PHA-665752-induced apoptosis in MET-activated gastric cancer cells.

### Cell-derived IL-6 activates STAT3 via JAK in gastric cancer cells without MET activation

**Figure 4:**
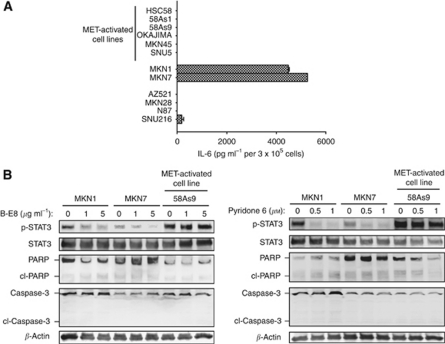
Effects of an antibody to IL-6 and a JAK inhibitor on cell signalling and survival in gastric cancer cell lines. (**A**) The indicated cell lines were cultured overnight in medium containing 10% serum and then incubated for 24 h in serum-free medium, after which the latter culture supernatants were collected and assayed for IL-6 with an ELISA. Data are means±s.d. from three independent experiments. (**B**) Cells were incubated in medium containing 10% serum for 72 h in the absence or presence of either the BE-8 antibody to IL-6 (1 or 5 *μ*g ml^−1^) or the JAK inhibitor pyridone 6 (0.5 or 1 *μ*M). Cell lysates were then subjected to immunoblot analysis with antibodies to the indicated proteins.

MET was found not to be activated in MKN1 and MKN7 cells despite the activation of STAT3 apparent in these cells. We therefore next investigated the mechanism by which STAT3 is activated in such cells. The IL-6 receptor shares the gp130 signalling component with other cytokine receptors, with activation of gp130 leading to activation of the tyrosine kinase JAK and subsequent phosphorylation of STAT3 (Kishimoto, 2005). To examine whether IL-6 contributes to STAT3 activation in gastric cancer cells, we first measured the secretion of IL-6 from such cells with the use of an ELISA. We found that both MKN1 and MKN7 cells released large amounts of IL-6 into the culture medium, whereas the secretion of IL-6 was virtually undetectable for the other 10 cell lines studied (Figure 4A). To determine whether cell-derived IL-6 indeed activates STAT3 through the IL-6–JAK–STAT3 pathway, we examined the effects of B-E8, anti-IL6 antibody, and the JAK inhibitor pyridone 6 on STAT3 phosphorylation. We found that both B-E8 and pyridone 6 inhibited the phosphorylation of STAT3 in MKN1 and MKN7 cells (which secrete IL-6) but not in MET-activated 58As9 cells (which do not secrete IL-6; Figure 4B). These results thus suggested that cell-derived IL-6 induces STAT3 activation through JAK in a subset of gastric cancer cells without MET activation.

### STAT3 activated by IL-6 contributes to migration of and invasion by gastric cancer cell lines

**Figure 5:**
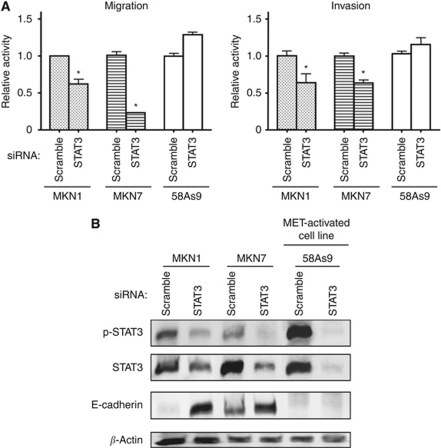
Effects of depletion of STAT3 on cell migration and invasion in gastric cancer cell lines. (**A**) The indicated cell lines were transfected with nonspecific (scramble) or STAT3 siRNAs for 24 h, isolated by exposure to trypsin, resuspended in serum-free medium, and transferred to the upper chamber of a Transwell or Boyden apparatus for measurement of cell migration or invasion, respectively. Cells that had migrated through or invaded the filter to reach the lower surface were fixed, stained, and counted by light microscopy at × 40 magnification. Data are expressed relative to the corresponding value for cells transfected with the control siRNA and are means±s.d. from three independent experiments. ^*^P<0.05 *vs* the corresponding value for control cells. (**B**) Cells were transfected with nonspecific (scramble) or STAT3 siRNAs for 48 h, after which cell lysates were subjected to immunoblot analysis with antibodies to the indicated proteins.

Given the role of STAT3 in MET-TKI-induced apoptosis in MET-activated cells (Figure 3), we investigated the effect of inhibition of STAT3 activated via the IL-6–JAK pathway on apoptosis. Although both B-E8 and pyridone 6 inhibited STAT3 phosphorylation, neither agent induced apoptosis in MKN1 or MKN7 cells (Figure 4B). To investigate other potential roles for STAT3 in these cells, we examined cell migration with a Transwell assay as well as cell invasion with a Boyden chamber assay. We found that depletion of STAT3 by RNAi inhibited the migration and invasion activities of MKN1 and MKN7 cells, both of which produce IL-6, without affecting those of MET-activated 58As9 cells, which do not express IL-6 (Figure 5A). These results suggested that STAT3 activated by IL-6 contributes to the migration of and invasion by MKN1 and MKN7 cells. Finally, to investigate the mechanism by which STAT3 depletion inhibits cell motility, we examined the expression of E-cadherin, a key component of epithelial cell adherence junctions that restrains both cell migration and invasion (Gumbiner, 1996; Wong and Gumbiner, 2003; Rivat *et al*, 2004; Andersen *et al*, 2005; Ramis-Conde *et al*, 2008). Depletion of STAT3 resulted in upregulation of E-cadherin expression in MKN1 and MKN7 cells but not in 58As9 cells (Figure 5B), consistent with the notion that STAT3 promotes cell migration and invasion through regulation of E-cadherin expression in gastric cancer cells in which STAT3 is activated by cell-derived IL-6.

## Discussion

We have here shown that STAT3 is activated at various levels in a subset of human gastric cancer cell lines. The activation of STAT3 did not correlate with the overall amount of the protein in these cells, indicating that STAT3 activity is dependent more on direct regulation than on protein abundance. Amplification of *MET* is often responsible for the activation of MET signalling, with such amplification occurring most frequently (10–20%) in gastric cancer (Kuniyasu *et al*, 1992; Nessling *et al*, 1998; Sakakura *et al*, 1999). In the present study, we found that all MET-activated cell lines manifested STAT3 phosphorylation and that PHA-665752 and a MET siRNA each inhibited the phosphorylation of STAT3 in these cells, indicating that STAT3 activation is closely linked to MET signalling in MET-activated gastric cancer cells. Signal transducer and activator of transcription 3 activation by various receptor tyrosine kinases has previously been shown to be JAK dependent or JAK independent (Olayioye *et al*, 1999; Paukku *et al*, 2000; Ren and Schaefer, 2002; Bong *et al*, 2007); however, it is unclear whether STAT3 is phosphorylated through JAK in MET-activated gastric cancer cell lines. Mutations in the kinase domain of the epidermal growth factor receptor result in STAT3 activation via the IL-6–JAK pathway in human lung adenocarcinoma (Gao *et al*, 2007). Activation of MET by hepatocyte growth factor has also been shown to induce IL-6 expression and subsequent STAT3 activation through JAK signalling in human liver cell lines (Lee *et al*, 2009). In the present study, we found that IL-6 was not secreted into the culture medium by, and that a JAK inhibitor had no effect on STAT3 phosphorylation in, MET-activated gastric cancer cells previously shown to have an increased *MET* copy number (Takada *et al*, 2005; Smolen *et al*, 2006; Okamoto *et al*, 2010), indicating that STAT3 phosphorylation is mediated by a JAK-independent mechanism in such cells.

Inhibition of STAT3 has been shown to reduce cell viability and to promote apoptosis in various cancer models (Buettner *et al*, 2002; Germain and Frank, 2007). We have now shown that forced expression of STAT3-CA attenuated PHA-665752-induced apoptosis in cells with MET activation, indicating that inhibition of STAT3 activity has a role in MET-TKI-induced apoptosis in MET-activated gastric cancer cells. Activated MET thus appears to contribute to cell survival, at least in part, through the activation of STAT3 in MET-activated gastric cancer cells. On the other hand, we found that gastric cancer cells in which STAT3 is activated in the absence of MET activation secreted IL-6, and this cytokine then activated STAT3 through the JAK pathway. With the use of an antibody to IL-6 and a JAK inhibitor, we also found that, in such cells, IL-6–JAK–STAT3 signalling did not appear to be required for cell survival. Furthermore, depletion of STAT3 by RNAi had no substantial effect on cell growth in MKN1 and MKN7 cells (data not shown). Signal transducer and activator of transcription 3 functions in the regulation of various cellular activities including migration and invasion in addition to proliferation and apoptosis (Rivat *et al*, 2004; Cheng *et al*, 2008; Senft *et al*, 2011). We found that depletion of STAT3 activated by cell-derived IL-6 inhibited cell migration and invasion in MKN1 and MKN7 cells, whereas depletion of STAT3 activated by MET had no such effects. These results suggest that STAT3 has different roles depending on the mechanism of its activation in gastric cancer cells. To investigate the mechanism by which STAT3 regulates cell migration and invasion, we examined whether RNAi-mediated depletion of STAT3 affected the expression of E-cadherin, which has an important inhibitory role in both migration and invasion (Gumbiner, 1996; Wong and Gumbiner, 2003; Rivat *et al*, 2004; Andersen *et al*, 2005; Ramis-Conde *et al*, 2008). We found that STAT3 negatively regulates E-cadherin expression in MKN1 and MKN7 cells, both of which produce IL-6, but not in MET-activated cells. The genes targeted by STAT3 for transcriptional regulation may thus depend on the mechanism of STAT3 activation or cell type. Further studies are required to determine the molecular underpinnings of such differential regulation.

In conclusion, we have shown that gastric cancer cell lines can be classified on the basis of the mechanism of STAT3 activation (induced by MET or IL-6) into distinct and non-overlapping subsets. Furthermore, activated STAT3 contributes to either survival or cell motility depending on the mechanism of its activation in gastric cancer cell lines. Strategies that target STAT3 signalling may thus provide novel approaches to gastric cancer intervention. Further studies are required to investigate whether the intrinsic biological properties of STAT3 are affected by differences in the mechanism of STAT3 activation.
